# Expression of Root-Related Transcription Factors Associated with Flooding Tolerance of Soybean (*Glycine max*)

**DOI:** 10.3390/ijms151017622

**Published:** 2014-09-29

**Authors:** Babu Valliyodan, Tara T. Van Toai, Jose Donizeti Alves, Patricia de Fátima P. Goulart, Jeong Dong Lee, Felix B. Fritschi, Mohammed Atiqur Rahman, Rafiq Islam, J. Grover Shannon, Henry T. Nguyen

**Affiliations:** 1 Division of Plant Sciences, University of Missouri, Columbia, MO 65211, USA; E-Mail: fritschif@missouri.edu; 2 United States Department of Agriculture-Agricultural Research Service, Soil Drainage Research Unit, Columbus, OH 43210, USA; E-Mail: tvantoai@yahoo.com; 3 Departamento de Biologia, Universidade Federal de Lavras, Lavras, MG 37200-000, Brazil; E-Mail: jdalves@ufla.br; 4 Centro Universitário de Lavras—UNILAVRAS, 37200-000, Lavras, MG 37200-000, Brazil; E-Mail: patriciagoulart@unilavras.edu.br; 5 Division of Plant Sciences, University of Missouri, Delta Center, Portageville, MO 68373, USA; E-Mails: jdlee@knu.ac.kr (J.D.L.); shannong@missouri.edu (J.G.S.); 6 Division of Plant Biosciences, Kyungpook National University, Daegu 702-701, Korea; 7 Office of Information Technology, Ohio State University, Columbus, OH 43210, USA; E-Mails: rahman.5@osu.edu (M.A.R.); islam.27@osu.edu (R.I.)

**Keywords:** abiotic stress tolerance, anaerobic genes, gene expression, hypoxia, waterlogging

## Abstract

Much research has been conducted on the changes in gene expression of the model plant *Arabidopsis* to low-oxygen stress. Flooding results in a low oxygen environment in the root zone. However, there is ample evidence that tolerance to soil flooding is more than tolerance to low oxygen alone. In this study, we investigated the physiological response and differential expression of root-related transcription factors (TFs) associated with the tolerance of soybean plants to soil flooding. Differential responses of PI408105A and S99-2281 plants to ten days of soil flooding were evaluated at physiological, morphological and anatomical levels. Gene expression underlying the tolerance response was investigated using qRT-PCR of root-related TFs, known anaerobic genes, and housekeeping genes. Biomass of flood-sensitive S99-2281 roots remained unchanged during the entire 10 days of flooding. Flood-tolerant PI408105A plants exhibited recovery of root growth after 3 days of flooding. Flooding induced the development of aerenchyma and adventitious roots more rapidly in the flood-tolerant than the flood-sensitive genotype. Roots of tolerant plants also contained more ATP than roots of sensitive plants at the 7th and 10th days of flooding. Quantitative transcript analysis identified 132 genes differentially expressed between the two genotypes at one or more time points of flooding. Expression of genes related to the ethylene biosynthesis pathway and formation of adventitious roots was induced earlier and to higher levels in roots of the flood-tolerant genotype. Three potential flood-tolerance TFs which were differentially expressed between the two genotypes during the entire 10-day flooding duration were identified. This study confirmed the expression of anaerobic genes in response to soil flooding. Additionally, the differential expression of TFs associated with soil flooding tolerance was not qualitative but quantitative and temporal. Functional analyses of these genes will be necessary to reveal their potential to enhance flooding tolerance of soybean cultivars.

## 1. Introduction

Flooding is a common environmental stress that impacts plant growth and reduces soybean grain yield in the humid temperate region of the United States, where heavy rainfall can exceed surface and subsurface drainage capabilities [1,2,3]. Flooding damage ranked second only to combined heat and drought stress in causing economic losses to U.S. agriculture over the 14-year period from 1990 to 2004 [4]. Given the current trend in global climate change with more extreme weather patterns, the National Aeronautics and Space Administration simulation models predicted that flooding damage to crops in the U.S. will double to ~$3 billion per year by the year 2030 [5].

Lack of oxygen has been proposed as the main problem associated with flooding. Indeed, tolerance to anoxia and hypoxia has been used synonymously with tolerance to flooding stress. During the last two decades, much research has been conducted on the molecular, biochemical and physiological responses of plants to the lack of oxygen, but relatively little information is available regarding the responses to soil flooding *per se* [6,7,8,9,10,11,12,13,14]. A significant change in the protein synthesis in roots occurs during anaerobiosis [15,16,17]. Most of the identified anaerobic proteins (ANPs) are enzymes involved in sugar metabolism, glycolysis and fermentation [18,19,20,21,22,23,24,25].

In addition, hypoxia induces genes coding for transcription factors [24,25,26,27], signal transduction components [28], non-symbiotic hemoglobin [29,30], as well as those involved in ethylene biosynthesis [31,32], nitrogen metabolism [10] and cell wall loosening [33,34].

Recent profiling of global gene expression by microarray technology revealed that about 5% to 14% of all the *Arabidopsis* genes examined are differentially expressed in response to low-oxygen stress [10,12,13,14,35,36,37]. Liu *et al.* [12] reported that 2085 genes in the whole-genome amplicon arrays of 26,777 genes showed significant differential expression to low-oxygen. These genes were involved in a wide range of biological processes, molecular functions, cellular components and metabolic pathways as identified by gene ontology analysis. Variability in global gene expression in response to oxygen availability has been reported across species, organ and developmental stage [38]. In an attempt to identify the core transcriptomic responses to anoxia, Narsai* et al.* [38] conducted three transcript profiling experiments with rice embryos and seedlings. Expression of as many as 9596 transcripts was found to change significantly in response to oxygen availability in at least one experiment. However, only 1866 transcripts showed significant changes across all experiments and organs. This set of core transcripts identified alterations in carbohydrate and lipid metabolism and putative regulatory mechanisms that allow rice to grow under anaerobic conditions [38]. Comparative transcriptome analyses of the submergence-tolerant M202 (*Sub1*) rice and its intolerant M202 isoline by Jung* et al.* [39] identified 898 genes that were differentially expressed in response to submergence. These genes were associated with a number of pathways including anaerobic respiration, hormone responses, and antioxidant systems governing the tolerance response. Large-scale transcriptome analyses of the low-oxygen responses in 21 organisms from four kingdoms—Plantae, Animalia, Fungi, and Bacteria—indicated that the responses in metabolic pathways associated with glycolysis, fermentation, alternative respiration, metabolite transport, reactive oxygen species amelioration, chaperone activity, and ribosome biogenesis were highly conserved, while the responses of genes involved in signaling and transcriptional regulation were poorly conserved across kingdoms [40]. Similarly, low-oxygen stress induced the expression of transcripts associated with energy yielding pathways—glycolytic flux, ethanol fermentation, starch and sucrose degradation—in the tolerant gray poplar (*Populus x canescens*) while it down-regulated those involved in energy demanding processes [41]. High-throughput analysis of gene responses to flooding and low oxygen stress in soybean has lagged behind that of other organisms.

Soybeans are known to be sensitive to flooding stress. Flooding can reduce soybean yield by 17% to 43% at the vegetative growth stage and by 50% to 56% at the reproductive stage [42]. However, genetic variability for flooding tolerance exists among soybean cultivars [2]. Since current U.S. soybean cultivars come from a narrow genetic base [43,44,45], soybeans with better soil flooding tolerance may be found in cultivars and landraces from other countries [46,47].

Previously, Shannon* et al.* [48] reported on screening more than 300 soybean plant introductions from the United State Department of Agriculture (USDA), Soybean Germplasm Collection (Urbana, IL, USA) for flooding tolerance. Among the tolerant genotypes of interest, PI408105A showed only 32.1% reduction in yield under five days of flooding compared to 81.2% reduction in the flooding sensitive breeding line S99-2281. Based on these results, PI408105A and S99-2281 were selected for more detailed examination of characteristics resulting in the differential flooding tolerance.

This study was conducted to investigate root growth, cellular responses and expression of root related transcription factors (TFs) of PI408105A compared to S99-2281 in response to soil flooding. Transcription factors are proteins that bind to specific DNA sequences (motifs) in the promoter regions of the genes to control their expression. Thus, TFs play an essential role in the up- or down-regulation of gene expression associated with the flood-tolerance response. In rice, the two transcription factors “ethylene response factors” (ERFs) at the *Sub1* locus are crucial for tolerance to complete submergence [49]. Transgenic “japonica” rice containing the *Sub1* locus was more tolerant to submergence than wild-type rice. Furthermore, over expression of *HRE1*, a hypoxia-inducible ERF of the same family as the *Sub1A*, in flood-intolerant *Arabidopsis* plants enhances their tolerance to anoxia [50]. Expression levels of one of the TFs from the NF-YA family, NF-YA10 increases in response to abiotic stresses, including low O_2_. It was reported that the overexpression of Arabidopsis NF-YA family members significantly reduced plant rosette size, delayed senescence and improved the submergence tolerance [51]. The improved tolerance was associated with enhancing anaerobic gene expression and ethanolic fermentation. Recently, Banti* et al.* [52] reported that over expression of the heat shock transcription factor *HsfA2* enhances the tolerance of transgenic *Arabidopsis* plants not only to heat but also to anoxia and submergence stress. Identification of additional TFs and understanding their molecular mechanism and function could enhance development of cultivars that have the ability to overcome flooding stress for the benefit of soybean producers, especially in regions where soil drainage is either impractical or impossible.

## 2. Results and Discussion

### 2.1. Physiological, Morphological and Anatomical Mechanism of Flooding Tolerance

#### 2.1.1. Root Growth

**Figure 1:**
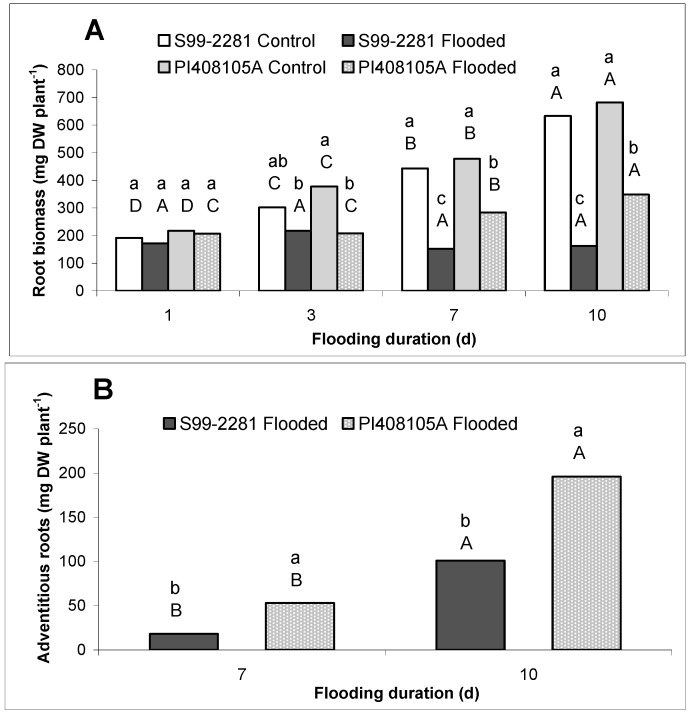
Root growth as measured by seminal root biomass (**A**) and adventitious root biomass (**B**) of control and flooded PI408105A and S99-2281 plants. Lower-case letters denote comparison between genotype/treatment at the same flooding duration (in days). The upper-case letters denote comparison within each genotype/treatment across different flooding duration. Means at each flooding duration with the same lower-case letter were not significantly different at *p* < 0.05. Means of each genotype/treatment with the same upper-case letters were not significantly different across different flooding duration.

Flooding for one day did not affect root biomass of each genotype (Figure 1A). Root biomass of S99-2281 plants remained unchanged during the entire 10 days of flooding. However, roots of PI408105A plants increased 31 mg·DW·day^−^^1^ between the 3rd and 7th day of flooding and 43 mg·DW·day^−^^1^ between the 7th and 10th day of flooding. Adventitious roots formed on the submerged stem above the soil surface and were visible beginning on the 3rd day of flooding in PI408105A, and one day later in S99-2281 plants. The adventitious root biomass produced by PI408105A was 2.8 and 1.9 times greater than that produced by S99-2281 at the 7th and 10th days of flooding, respectively (Figure 1B). Control plants of both genotypes did not produce adventitious roots.

**Figure 2:**
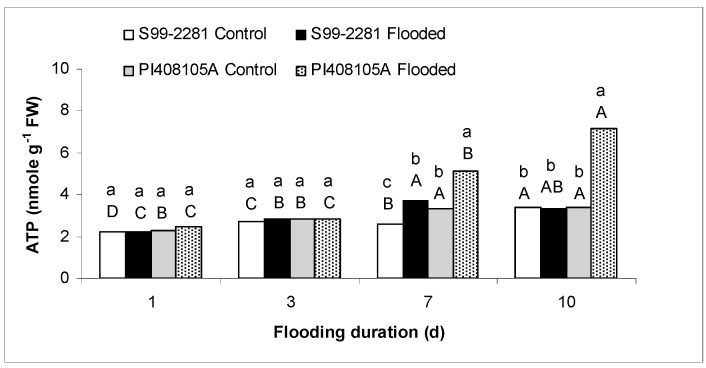
Root ATP concentration of control and flooded PI408105A and S99-2281 plants. Lower-case letters denote comparison between genotype/treatment at the same flooding duration (in days). The upper-case letters denote comparison within each genotype/treatment across different flooding duration. Means at each flooding duration with the same lower-case letter were not significantly different at *p* < 0.05. Means of each genotype/treatment with the same upper-case letters were not significantly different across different flooding duration.

Both genotypes were negatively affected by flooding at the one-leaf, early vegetative V1 stage. However, the effects of flooding were more severe in S99-2281 than PI408105A, confirming the results obtained from field-based screening efforts [48]. Roots of S99-2281 plants had less biomass than roots of the tolerant PI488105A plants, especially at the 10th day of flooding (Figure 1A and Figure 2). Additionally, solute leakage was also lower from PI408105A roots at the 10th day of flooding than from S99-2281 roots (data not shown). These results revealed that adaptation to flooding stress occurred earlier and was more pronounced in the flood tolerant genotype than in the flood sensitive genotype.

#### 2.1.2. Root ATP Concentration

No significant differences were observed among genotypes and treatments at the first two sampling dates (Figure 2). However, 7 days into flooding, root ATP concentration was greater in flooded plants of both genotypes compared to control plants (*p* ≤ 0.05). At 10 days of flooding, roots of flooded PI408105A plants contained significantly greater levels of ATP than roots of control plants (*p* ≤ 0.05). The ATP concentrations of S99-2281 roots were not different between flooded and control plants, nor did they differ from ATP concentration in control PI408105A roots. The high ATP concentration in PI408105A roots on the 7th and especially on the 10th day of flooding was associated with a large amount of fleshy adventitious roots and new basal roots produced above and below the soil surface.

#### 2.1.3. Root Aerenchyma

**Figure 3:**
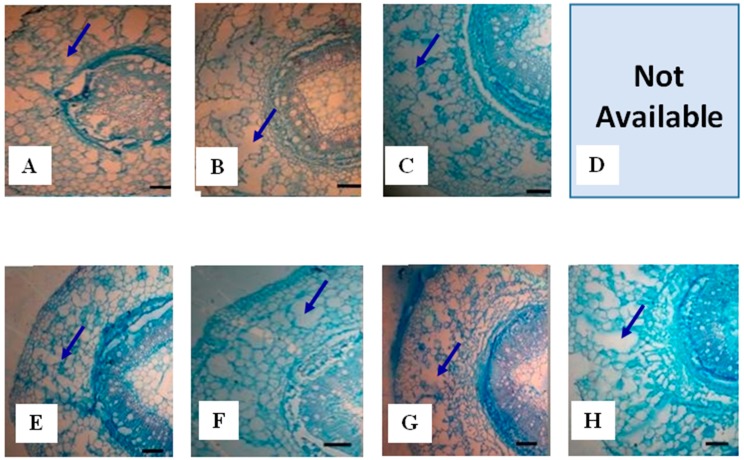
Aerenchyma development in roots of PI408105A (**A**–**D**) and S99-2281 plants (**E**–**H**) at one day (**A** and **E**), three days (**B** and **F**), seven days (**C** and **G**), and ten days (**D** and **H**) of soil flooding. Reproducible photomicrographs of PI408105A roots at the 10th day flooding treatment (**D**) were not available due to damages caused by sectioning of this highly porous segment. The scale bar is 100 μm.

Aerenchyma development in roots of PI408105A and S99-2281 is reported in Table 1 and Figure 3. Roots of both genotypes grew larger in diameter in response to flooding stress. Total root cross-sectional area, central cylinder area and cortex area were larger in S99-2281 than in PI408105A roots at all four sampling dates. Aerenchyma occupied a larger area of the cross section with longer flooding duration. While there was no difference in absolute aerenchyma area between the genotypes, the relative area of aerenchyma in the root cross section and in the cortex was significantly larger (*p* ≤ 0.05) in PI408105A roots than in S99-2281 roots. Reproducible photomicrographs of the root section of PI408105A at the 10th day flooding treatment were not available due to damages caused by sectioning of this highly porous segment.

**Table 1:** Aerenchyma development in roots of PI408105A and S99-2281 at 1, 3, 7 and 10 days of flooding. FD: Flooding duration, TRA: Total root cross section area, CCA: Central cylinder area, COA: Cortex area, TAA: Areas of aerenchyma, PAA: Percentage of aerenchyma area in the root cross-section, PAC: Percentage of aerenchyma area in the cortex. The mean of each root was calculated from four representative sections; the mean of each replicate was calculated from the two roots in each pot. The treatment mean of three biological replicates was computed and separated by the Skott-Knott test at *p* < 0.05 (the significance levels a–d are shown).

Genotype	FD (day)	TRA (mm^2^)	CCA (mm^2^)	COA (mm^2^)	TAA (mm^2^)	PAA (%)	PAC (%)
PI408105A	1	102.5 d *	11.2 d	91.3 c	15.39 c	15.1 b	16.9 b
PI408105A	3	129.9 d	24.5 c	105.5 c	26.64 b	20.5 a	25.2 a
PI408105A	7	171.0 b	32.9 b	138.2 b	30.90 a	18.2 a	22.6 a
PI408105A	10	-	-	No results *	-	-	-
S99-2281	1	150.1 c	28.2 b	121.9 b	13.28 c	8.9 c	11.0 c
S99-2281	3	166.9 b	29.6 b	137.2 b	25.07 b	15.1 b	18.3 b
S99-2281	7	213.9 a	48.7 a	165.2 a	33.94 a	15.9 b	20.6 b
S99-2281	10	180.8 b	25.2 c	155.6 a	23.01 b	12.9 b	14.9 b

* No reproducible data were obtained because the samples were damaged by sectioning due to the large area of aerenchyma.

Roots of PI408105A developed aerenchyma earlier and more profusely in roots of S99-2281 (Figure 3 and Table 1). Greater access to oxygen as a result of earlier aerenchyma formation and increased adventitious and basal root production likely resulted in the higher ATP concentration in PI408105A roots than S99-2281 roots. These adaptive responses probably played important roles in allowing roots of the flood-tolerant genotype to resume biomass accumulation between the 3rd and 7th days of flooding (Figure 1A). The quicker response of tolerant soybean genotypes to flooding compared to susceptible genotypes has been reported previously in a study using an image processing technique to monitor leaf movement [53]. The importance of aerenchyma and adventitious roots in flooding tolerance responses has been shown in many monocotyledonous crop species including rice [54,55], maize [56,57], wheat [58], and barley [59,60], as well as dicotyledonous species such as alfalfa [61] and soybean [62,63]. Treatment with inhibitors to prevent aerenchyma formation reduced plant tolerance to flooding stress [56,64].

### 2.2. Expression of Root-Related Transcription Factors and Anaerobic Genes

**Figure 4:**
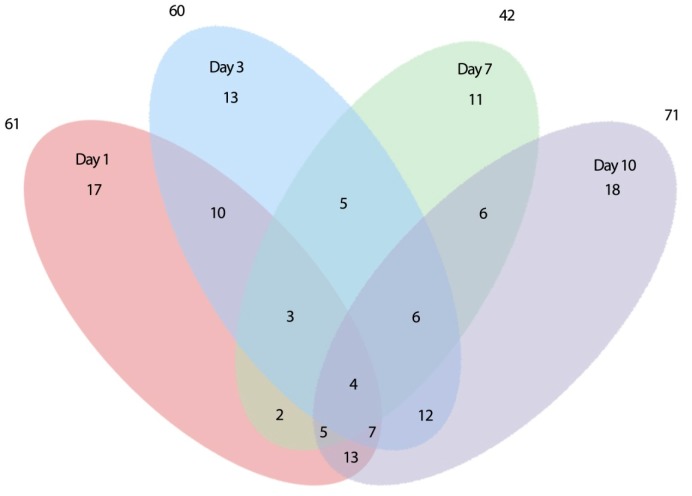
Venn diagram showing the number of differentially expressed genes (*p* < 0.05) between the two genotypes, whose expression was either unique or overlapping at 1, 3, 7 and 10 days of soil flooding stress.

The qRT-PCR experiment was conducted with primers for 192 genes, of which 165 genes were amplified successfully. Analysis of variance revealed that 130 genes were differentially expressed and that the differences in expression were highly significant among treatments, flooding durations, genes, and their interactions (Table S1). While the difference in expression between the genotypes was not significant, the interactions between genotype x genes, genotype x treatment, and genotype x flooding duration were significant (Table S2). Various expression patterns were observed for the 130 genes differentially expressed due to flooding at one or more time points. A summary of numbers of genes differentially expressed between the two genotypes over the course of the experimental duration is provided in a Venn diagram (Figure 4). This diagram visualizes a complex pattern of temporal gene expression including genes which expressed (a) uniquely at one time point; (b) at two time points; (c) at three time points; and (d) at all four time points of flooding. The 61 genes whose expression was significantly different between the two genotypes at the first day of flooding can be divided into eight groups based on the temporal expression pattern: (a) seventeen genes with a significant differential expression only at the 1st day of flooding; (b) ten genes with a significant differential expression at the 1st and 3rd days of flooding; (c) two genes with a significant differential expression at the 1st and 7th days of flooding; (d) five genes with a significant differential expression at the 1st and 10th days of flooding; (e) three genes with a significant differential expression at the 1st, 3rd and 7th days of flooding; (f) seven genes with a significant differential expression at the 1st, 3rd and 10th days of flooding; and (h) four genes with a significant differential expression at all four time points (1st, 3rd, 7th and 10th day of flooding; Figure 4).

#### 2.2.1. Early Differentially Expressed Genes between the Two Genotypes

**Figure 5:**
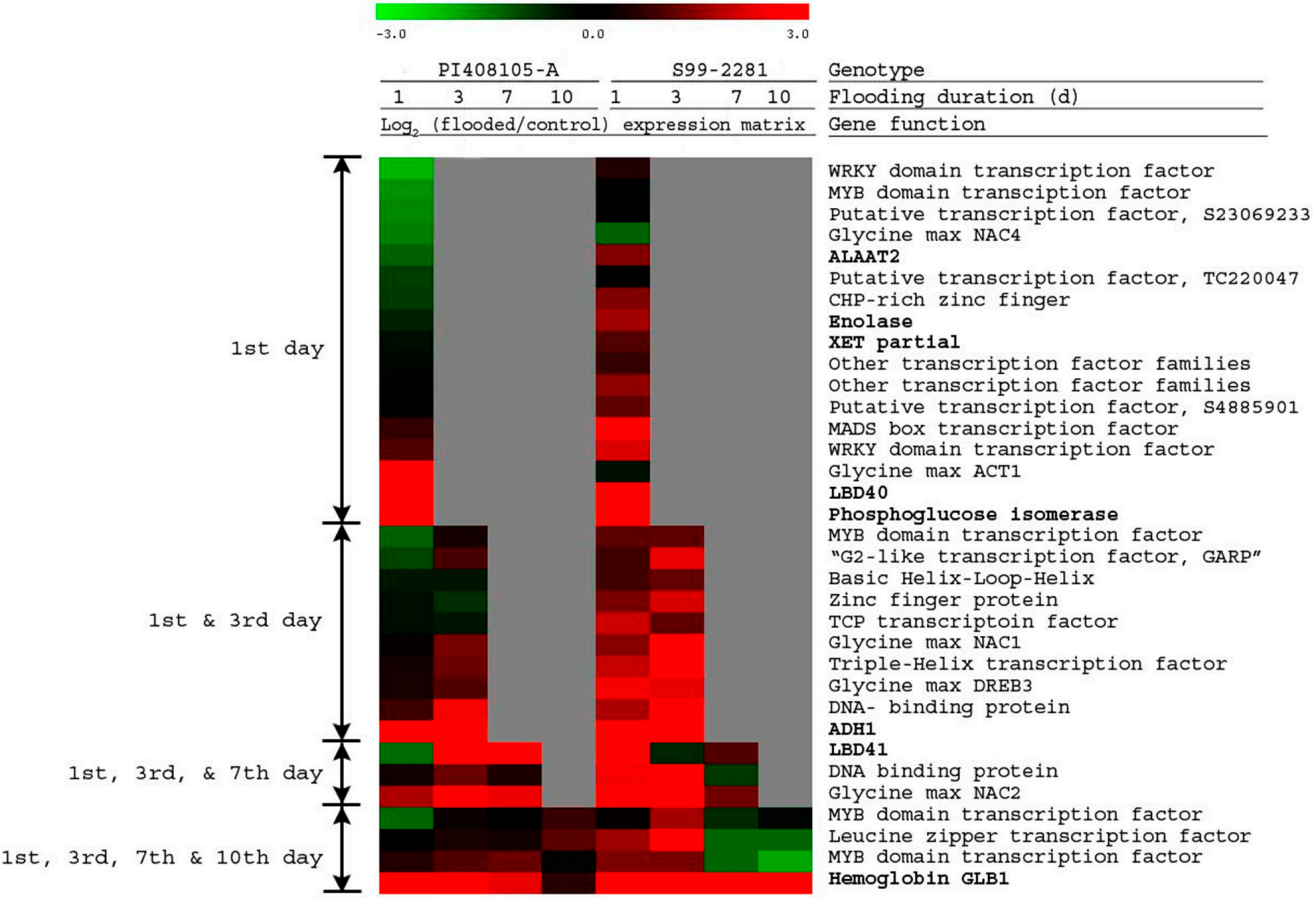
Expression matrix of genes that were differentially expressed in roots of PI408105A and S99-2281 genotypes at the 1st day (17 genes), the 1st and 3rd days (10 genes), the 1st, 3rd and 7th days (three genes), and the 1st, 3rd, 7th and 10th days (four genes) of soil flooding stress. The red color indicates genes up-regulated by flooding. The green color indicates down-regulation. Gray color indicates no significant difference in expression. Gene function in bold type indicates known anaerobic genes. The scale above the expression image shows the log_2_ (flooding/control).

The 17 genes differentially expressed between the two genotypes only at the 1st day of flooding included five known anaerobic genes: alanine amino transferase2 (*ALAAT2*), enolase, xyloglucan endotransglycosylase (*XET* partial), *LBD40*, and phosphoglucose isomerase; a soybean actin related gene (*ACT1*); and 11 TFs (Figure 5). The TFs included two WRKY, one MYB, one zinc-finger, one MADS box, one NAC domain, and five putative or unknown genes. Expression of the genes in this group of 17 genes was either unchanged or decreased in PI408105A roots, while in S99-2281 roots, expression was induced. The only exception was the soybean *ACT1,* which had higher expression in PI408105A (log_2_ = 4.05) than in S99-2281 roots (log_2_ = −0.38) (Figure 5).

The ten genes that were differentially expressed between the two genotypes at the 1st and 3rd days of flooding included the *ADH1* gene, a known anaerobic gene. The other nine genes were TFs, which included the Dehydration-Responsive Element Binding gene 3 (*DREB3*) and the soybean *NAC1* domain gene. Expression of genes in this group was lower in PI408105A roots than in S99-2281 roots (Figure 5).

The three genes that differentially expressed during the first three time points (1st, 3rd and 7th day) of flooding, included *LBD41*, the soybean *NAC2* transcription factor and a DNA binding protein. At the 1st day of flooding, expression of the genes in this group was lower in PI408105A roots than in S99-2281 roots. Expression increased in PI408105A roots at the 3rd day and remained high at the 7th day of flooding, but decreased to lower levels in S99-2281 roots at this late time point.

**Figure 6:**
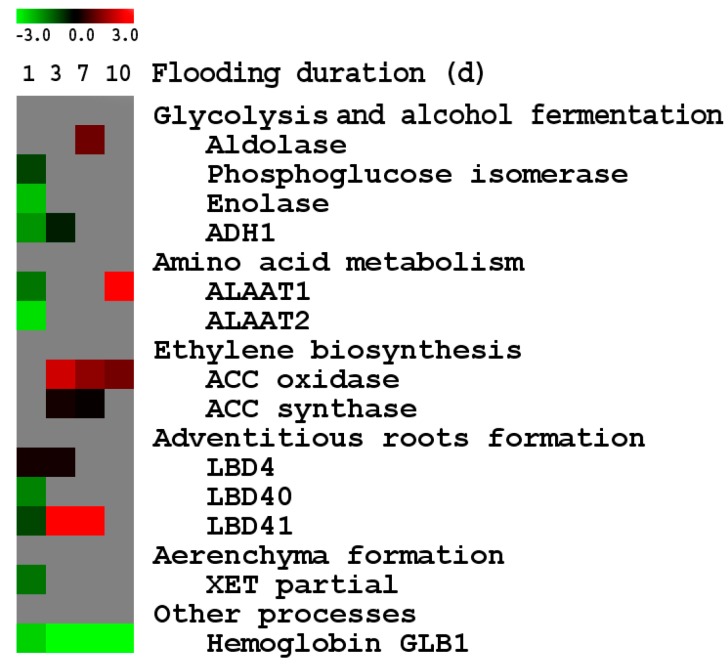
Expression of known anaerobic genes in roots of PI408105A relative to S99-2281 roots at 1, 3, 7 and 10 days of soil flooding stress. The red color indicates that expression in PI408105A roots was higher than in S99-2281 roots. The green color indicates that expression in PI408105 roots was lower than in S99-2281 roots. The scale above the expression image shows the ratio in log_2_ (flooding/control) of PI408105A relative to S99-2281 roots.

Of the four genes which were differentially expressed between the two genotypes at all four time points of flooding stress, one gene was the haemoglobin *GLB1* gene. The other three genes were transcription factors (Figure 5): two MYB domain transcription factors (S4877491 and S4910460) and a leucine zipper transcription factor (S23061205). The *GLB1* gene was induced at the 1st day of flooding to a very high level in S99-2281 roots (log_2_ = 5.44) and remained high throughout the 10 days of flooding (Figure 6). The induction was lower in PI408105A roots at the 1st day of flooding (log_2_ = 2.97) and was further reduced at day 7 and day 10 of flooding. Expression of the leucine zipper transcription factor gene was induced to much higher levels at days 1 and 3 of flooding in S99-2281 roots (log_2_ = 1.60 and 2.88, respectively) compared to PI408105A roots (log_2_ = −0.13 and 0.46, respectively). However, expression was reduced significantly in S99-2281 roots at days 7 and 10 of flooding (log_2_ = −1.31 and = −1.29, respectively) while it increased in PI408105A roots (log_2_ = 1.07) at day 10 of flooding. Similar temporal expression patterns that showed induction at the 7th and 10th day of flooding in PI408105A roots and suppression in S99-2281 roots, were also detected in the two MYB domain transcription factors (Figure 5).

#### 2.2.2. Differential Expression of Known Anaerobic Genes between the Two Genotypes

Of the 18 known anaerobic genes included in the study, 13 genes were differentially expressed between the two genotypes at one or more time points (Figure 6). Of these, seven genes—*ADH1*, *LBD40*, *XET* partial, enolase, alanine amino transferase 2, phosphoglucose isomerase, and hemoglobin *GLB1—*expressed higher in S99-2281 than in PI408105A roots. The other six genes—alanine amino transferase 2, *LBD41*, *LBD4*, *AAC* oxidase, *ACC* synthase and aldolase—either expressed higher or their expression increased to higher levels in PI408105A roots with increasing flooding duration. In this study, expression of both *ADH1* and *ADH2* was induced at the 1st day of flooding as much as 64- to 256-fold compared to the control and remained high until the 10th day of flooding in both genotypes, but only the expression of *ADH1* gene at the 1st and 3rd days of flooding was significantly different between the tolerant and susceptible genotypes. Expression of a cytoplasmic aldolase gene, an enzyme of the glycolysis pathway, increased 19% in the tolerant genotype but declined nearly 50% in the susceptible genotype at the 7th day of flooding. Three members of the lateral organ boundary domain (*LBD*) gene family (*LBD40*, *LBD41* and *LBD4*) were differentially expressed between the two genotypes in response to flooding (Figure 6). Expression of the *LBD40* gene was induced to a higher level at the 1st day of flooding in S99-2281 than in PI408105A roots. The induction remained high but no differences were observed in either genotype as flooding progressed. Expression of *LBD41* was induced earlier (at the 3rd day of flooding) and remained higher at the 7th day of flooding in PI408105A roots compared to S99-2281 roots. The induction of *LBD4* expression was also higher in PI408105A than in S99-2281 roots. The expression of *ACC* synthase and oxidase genes was much higher in PI408105A roots than in S99-2281 roots in response to flooding.

#### 2.2.3. Gene Expression Associated with Flooding Tolerance

To gain an understanding of gene expression associated with the tolerance response of PI408105A to soil flooding, high-throughput qRT-PCR was conducted with 169 root-related transcription factor genes. In addition, 18 known anoxia-induced genes were added to the study to verify the flood-tolerance responses of these roots. In this study, 80% of the 165 genes that were amplified showed changes in expression at one or more time points of flooding stress (Figure 4; Table S1). It is interesting to note that this proportion was one order of magnitude higher than the 7.8% of genes in *Arabidopsis* plants that expressed differentially during the first 24 h of hypoxia as detected by the 26,777 whole-genome DNA amplicon microarrays [12]. The high percentage of differentially expressed genes found in this soybean qRT-PCR study can be partially attributed to the known anaerobic genes that were included in the study. The results also revealed that root-related transcription factor genes were more affected by flooding stress than genes or gene amplicons reported in the microarray studies.

#### 2.2.4. Expression of Known Anaerobic Genes

Enhanced expression of genes in glycolysis and fermentation pathways have been shown to be associated with the tolerance of rice [39] to submergence, and of *Arabidopsis* [50] and gray poplar [41] to anoxia. In the present study, flooding induced the expression of glycolytic and ethanol fermentation genes in roots of both soybean genotypes. However, except for aldolase, the induction was higher in roots of the susceptible genotype (S99-2281) than in those of the tolerant genotype (PI408105A). The greater induction of phosphoglucose isomerase, enolase and *ADH1* genes in S99-2281 roots may reflect reduced formation of aerenchyma and adventitious roots in the susceptible genotype. Additionally, research in the last three decades has substantiated that, beyond a minimum level (*i.e.*, *ADH1*^−^ null mutants), there is no correlation between the level of *ADH* gene expression and tolerance to anoxia [65]. Over-expression of *ADH1* showed no enhancement in *Arabidopsis* plants’ tolerance to anoxia [11,66]. Among the genes that were induced to higher levels in PI408105A roots are two *LBD* genes. The *LBD* gene family coding for Lateral Organ Boundaries proteins are plant specific [67] and have been implicated in several development related functions including lateral root formation in *Arabidopsis* [68]. The greater induction of *LBD* genes might contribute to the earlier and more abundant adventitious root phenotype of the PI408105A than that of the S99-2281 plants.

The plant hormone ethylene has been implicated in the formation of adventitious roots and aerenchyma in maize [34]. Aminocyclopropanecarboxylate (ACC) synthase and ACC oxidase are the two enzymes of the ethylene biosynthesis pathway [69]. The greater and earlier induction of ACC synthase and ACC oxidase expression in PI408105A roots may also contribute to the early formation of adventitious roots and aerenchyma of the tolerant phenotype.

Low-oxygen stress increases the expression of the non-symbiotic hemoglobin GLB1 mRNA and protein in *Arabidopsis* roots and shoots [70]. Overexpression of the *GLB1* gene has been shown to enhance the survival of hypoxic stress in *Arabidopsis* [70]. In this study, hemoglobin *GLB1* expression levels were higher in S99-2281 roots than in PI408105A roots throughout the ten days of flooding. Expression of the *GLB* genes may be associated with anaerobiosis that was more prevalent in S99-2281 roots than in PI408105A roots based on the aerenchyma, adventitious roots and ATP concentration results.

#### 2.2.5. Flooding-Tolerance Candidate Genes

The changes in gene expression identified in this study can be attributed to either the adaptive responses leading to the formation of aerenchyma and adventitious roots or the deleterious side-effects of flooding stress. Since transcription factors can act as repressors or inducers of gene expression, flooding tolerance candidate genes may be either negatively or positively expressed in response to flooding. It seems likely that the genes that were differentially expressed on the 1st day of flooding and continued to be differentially expressed between the two genotypes at later time points may play a role in the tolerance responses. Among these were three NAC domain transcription factors, a cysteine, histidine and proline (CHP)-rich zinc finger gene and the soybean actin-related *ACT1* gene. The CHP-rich zinc finger is a TF containing a DNA binding and a calcium binding domain. Its homolog in *Arabidopsis*, AT2G37810, is induced by salt stress [71]. The soybean actin-related *ACT1* gene was the only gene with a much greater induction in PI408105A roots compared to S99-2281 roots. Three members of the plant-specific NAC transcription factor family—ANAC019, ANAC055 and ANAC072 were induced by abscisic acid drought, and salinity [72]. Transgenic plants overexpressing these NAC domain TFs were more tolerant to drought stress [72]. More recently, low-oxygen stress (0.1%) has been shown to induce the expression of the NAC domain transcription factor *ANAC102* in *Arabidopsis* roots, shoots and germinating seeds [14]. Expression of the *ANAC102* gene is important for the survival of germinating *Arabidopsis* seedlings following low-oxygen treatment [14]. Recently, several root related and water stress related NAC TFs were cloned from soybean [73].

Of the three TF genes with consistently different expression between the two genotypes at all four time points, the leucine zipper S23061205 has been annotated to be induced in soybean roots by drought stress [74]. Sequence homology searches of the *Arabidopsis* TAIR BLAST 2.2.8 database showed that this gene has high homology to At1g72040 (*E* value = 0.012), which codes for a nucleus located ATP-binding phosphotransferase enzyme involved in the nucleic acid metabolic process. Induced by cytokinin, At1g72040 has been shown to be involved in the cytokinin signaling pathway [75]. Interestingly, this leucine zipper S23061205 also shares sequence homology with At5g27760 (*E* value = 0.18), which was classified as “a hypoxia-responsive family protein”. The second TF in this group is the MYB domain S487749 gene, which showed very high sequence homology with At3g11450, a DNAJ heat shock *N*-terminal domain-containing protein (*E* value = 5 × 10^−14^). The DNAJ heat shock protein functions in coordinating stress responses through protein folding for translocation across organelle membranes. The third TF, a MYB-domain transcription factor S4910460, showed sequence homology with the *Arabidopsis* gene At5g47390 (*E* value = 0.069). At5g47390 is one of the 29 *Arabidopsis* transcription repressors recently identified in *Arabidopsis* [76]. Expression of this gene is responsive to salt stress and cadmium ion toxicity as well as to many plant hormones including abscisic acid, ethylene, gibberellin, jasmonic acid, and salicylic acid [77].

## 3. Experimental Section

### 3.1. Plant Materials and Flooding Treatment

A factorial combination experiment of 2 soybean genotypes x 2 flooding durations was set-up using a randomized complete block design with three replications for each treatment combination. Seeds of PI408105A and S99-2281 were planted in 3.8-L pots filled with autoclaved top-soil from a Mexico Silt Loam (fine, smectic, mesic, Vertic Epiaqualf) at 4 seeds per pot. After emergence, seedlings were thinned to two plants per pot. Plants were grown in a greenhouse without supplemental light. Average daily maximum temperature was 29.5 °C and average daily minimum temperature was 15.8 °C over the course of the growth period.

Flooding stress was imposed at the V1 (vegetative stage with first trifoliolate—one set of unfolded trifoliolate leaves) growth stage [78] by placing individual pots into 19-L tubs (lined with white high-density polyethylene plastic sheet to eliminate water drainage) and adding water to a level 3 cm above the soil surface. Plants in the control treatment were also placed into 19-L tubs but without the plastic liner and only watered to maintain normal growth without stress. The experimental design was a randomized complete block with three replications. Root measurements and samples were taken at 1, 3, 7 and 10 days of flooding.

### 3.2. Phenotypic Analysis of Responses to Flooding

#### 3.2.1. Determination of Root Biomass

Plants were cut at the soil surface and roots were carefully removed from pots and washed by gentle agitation in a large, 19-L tub of water to remove all soil particles. Roots were quickly blotted dry with paper towels and weighed. Adventitious roots were only collected from the 7- and 10-day sampling time points of the flooded treatment where they were visible. After weighing, adventitious and seminal roots were combined and representative samples were collected for (1) ATP determination; (2) RNA extraction; and (3) dry weight determination by drying at 85 °C for 48 h in a forced air oven. Root samples for ATP assay and RNA extraction were immediately frozen in liquid nitrogen and kept at −80 °C until extraction. Root fragments (10 mm) from the upper-most part of the tap root were collected from each plant at all four time points of the flooding treatment for aerenchyma determination. Root dry weight was calculated from the fresh weight using the percent dry weight of the samples.

#### 3.2.2. Analysis of Root ATP Content

Frozen roots (one gram) were ground to powder in liquid nitrogen and extracted in 20 mL of 0.6 M trichloroacetic acid (TCA) at 4 °C for 30 min with occasional agitation. The samples were centrifuged at 18,000× *g* for 20 min at 4 °C and the supernatant was collected. ATP was quantified by the bioluminescence assay with the recombinant firefly luciferase and its substrate d-luciferin using the ATP determination kit (Molecular Probes, Inc., Eugene, OR, USA) following the manufacturer’s protocol. The luminescence produced at 560 nm wavelength, proportional to the amount of ATP in the sample, was quantified with a luminometer (Veritas Microplate Luminometer, Turner BioSystems, Sunnyvale, CA, USA). Luminescence was converted to nmoles of ATP per gram of root fresh weight (FW) using a standard curve of known ATP levels.

#### 3.2.3. Analysis of Aerenchyma

The root fragments were fixed for 72 h at room temperature in a solution of 5% (*v*/*v*) formaldehyde, 5% (*v*/*v*) glacial acetic acid, and 90% (*v*/*v*) 70% ethanol [79]. The specimens were preserved in 70% ethanol at room temperature until they were embedded in paraffin. Sections (5 μm thick) were obtained using a rotary microtome and were stained with safranin-blue astra (1% astra blue and 0.1% safranin) as described by [80]. Sections were visualized with a 4 × 0.16 numerical aperture (NA) UPlan Apochromat objective on an Olympus IX70 inverted microscope (Olympus Corporation, Tokyo, Japan). Color images were acquired with a monochrome CCD camera (Orca C4742-80-12AG, Hamamatsu, Japan) and a Micro*Color filter (CRi, Boston, MA, USA) controlled by Metamorph 4.6 software (Molecular Devices, Sunnyvale, CA, USA). Since the sections were larger than the field of view of the camera, multiple overlapping images of each section were taken and then merged together with the Photomerge function of Photoshop CS (Adobe, San Jose, CA, USA). The areas of the root cross section (TRC), central cylinder (CCA), cortex (COA), and aerenchyma (AEA) were measured for each of the four representative sections of each sample using the Image Tool software by University of Texas Health Science Center, San Antonio, TX, USA. The percentage of aerenchyma area in the root cross-section (PAR) and percentage of aerenchyma area in the cortex (PAC) were calculated.

#### 3.2.4. Statistical Analysis

The root biomass and ATP data were analyzed by ANOVA and Duncan’s multiple range test at *p* ≤ 0.05. For anatomical traits, the mean of each root was calculated from four representative sections; the mean of each replicate was calculated from the two roots in each pot. The treatment mean of three biological replicates was computed and separated by the Skott-Knott test at *p* ≤ 0.05.

### 3.3. High Throughput Quantitative Reverse-Transcription PCR (qRT-PCR)

#### 3.3.1. RNA Isolation, DNase Treatment and cDNA Synthesis

Total RNA was isolated using TRIZOL reagent (Invitrogen, Carlsbad, CA, USA) according to the protocol provided by the manufacturer. RNA concentration and integrity were measured prior to DNase digestion with the NanoDrop ND-1000 UV-Vis spectrophotometer (NanoDrop Technologies, Wilmington, DE, USA). To remove genomic DNA contamination, each sample of ~30 μg of total RNA was digested with Turbo DNA-free DNase I (Ambion, Foster City, CA, USA) according to the manufacturer’s instructions for routine DNase treatment. First-strand cDNA synthesis was performed using 25 μg of DNase-treated total RNA with reverse transcriptase and dNTPs (Promega, Madison, WI, USA) in a reaction volume of 20 μL according to the manufacturer’s protocol.

#### 3.3.2. Primers of Soybean Transcription Factors for qRT-PCR

The soybean genome was recently sequenced by the Department of Energy-Joint Genome Institute (DOE-JGI) and is publicly available. Mining of this sequence identified 5671 soybean genes as putative regulatory genes, including transcription factors [81]. A library of qRT-PCR primers was developed to allow for sensitive measurement of the expression of ~1200 different soybean transcription factors (25% of total soybean TF genes). All the primers were designed using the modified Primegene program. Primer sets were previously used to profile gene expression in various soybean tissues and under various biotic and abiotic stress conditions. From these comparisons 169 root related transcription factors were identified. The 169 root-related transcription factors as well as 18 flooding-related genes, and 5 housekeeping genes were employed for qRT-PCR in this study (Table S3).

#### 3.3.3. qRT-PCR

qRT-PCR was conducted with cDNA samples from roots of three independent biological replicates for each treatment time point (a total of 24 samples). Reactions were performed in a 384-well plate format using the ABI 7900 HT Sequence Detection System (Applied Biosystems, Foster City, CA, USA) and SYBR Green PCR Master Mix (Applied Biosystems). Primer sets (0.2 μM final concentrations for each primer) and 3 μL cDNA SYBR Green mix were used in a final volume of 5 μL per well. The thermal profile of the qRT-PCR reactions was 50 °C for 2 min, 95 °C for 10 min, and 40 amplification cycles of 95 °C for 15 s and 60 °C for 1 min. The raw data was analyzed with the ABI-SDS 2.2.1 software package (Applied Biosystems) using an Rn threshold of 0.1 with automatic background subtraction to obtain the cycle threshold (*C*_t_) values. Linear regression analysis was performed to determine polymerase chain reaction efficiency from the given slope generated in the SDS 2.2.1 software (Applied Biosystems) with the LinRegPCR software [82,83]. For confirmation of primer specificity, the dissociation curves were verified.

#### 3.3.4. Statistical Analysis and Data Presentation

To evaluate the effects of flooding on expression of transcription factors of soybean genotypes over time, the data were statistically analyzed for multi-factorial analysis of variance using the statistical analysis software (SAS). The flooding, genotype, and flooding duration were considered as fixed factors and the block was considered as a random factor. Simple and interactive effects of predictor variables on the treatment means of the dependent variables were separated by the Duncan’s multiple range test at *p* < 0.05 unless otherwise mentioned. For anatomical traits, the mean of each root was calculated from four representative sections; the mean of each replicate was calculated from the two roots in each pot. The treatment mean of three biological replicates was computed and separated by the Skott-Knott test at *p* < 0.05.

The data were normalized to the expression of soybean ubiquitin gene (Gm*UBI*) which remained unchanged in response to flooding. The relative expression levels were calculated using the ∆∆*C*_t_ method [84]. The log_2_ ratio of expression of flooding to control samples was calculated for each gene. The logarithmic transformation was performed to ensure the up-regulated and down-regulated genes were distributed symmetrically around the *X*-axis [85]. The normalized expression data were analyzed statistically using the GLM procedure of SAS 9.1 for Windows (SAS Institute, Cary, NC, USA). Genes that were significantly different (*p* ≤ 0.05) between the genotypes due to flooding stress at each time point were identified. The expression ratio of the significant genes between the two genotypes was computed by subtracting the log_2_ (flooding/control) of S99-2281 from the log_2_ (flooding/control) of PI408105A. Gene expression was clustered and visualized using the TMEV module of the TM4 software developed originally by the Institute for Genomic Research, Rockville, MD, USA.

## 4. Conclusions

Controlled experiments on young soybean plants conducted under greenhouse conditions confirmed results from field studies that indicated greater flooding tolerance of PI408105A than S99-2281. Using high-throughput qRT-PCR we observed a complex gene expression pattern of TFs and known anaerobic genes associated with flooding tolerance responses. The results extend existing information on the molecular responses to anoxia and hypoxia to soil flooding stress *per se*. The differential gene expression associated with soil flooding tolerance was not qualitative but quantitative and temporal. Expression of genes of the ethylene biosynthesis pathway and the formation of roots was enhanced in the roots of the flooding tolerant PI408105A. Most specific candidates were two MYB domain TFs, one leucine zipper TF, and the *GLB1* hemoglobin gene, which were differentially expressed between the two genotypes during all four sampling times of the 10-day flooding period. Further functional analysis will evaluate the role of these genes in orchestrating the physiological, morphological and anatomical responses of the flooding tolerant soybean genotype.

## Supplementary Materials

Supplementary tables can be found at http://www.mdpi.com/1422-0067/15/10/17622/s1.

## Supplementary Files

Supplementary File 1
