# Supplementary material for: Expression of Root-Related Transcription Factors Associated with Flooding Tolerance of Soybean (Glycine max)
Source: Int J Mol Sci. 2014 Sep 29;15(10):17622–43. doi: 10.3390/ijms151017622 (PMC4227181; doi:10.3390/ijms151017622)
Supplement: Supplementary File 1 [file ijms-15-17622-s001.pdf]

Supplementary Information

Table S1. Log_2 (flooded/control) expression of genes whose expression was significantly different ($p \leq 0.05$) between the two genotypes in response to soil flooding at one or more time points. Blank cells indicate expression was not significant at $p \leq 0.05$.

Accession No.	Genotype	PI408105-A				S99-2281			
	**Flooding Duration (Day)**	**1**	**3**	**7**	**10**	**1**	**3**	**7**	**10**
	**Gene Function**	**Log_2 (Flooded/Control) Expression Ratio**							
	Transcription factors								
AY974349	Glycine max NAC1	0.212	1.297			1.426	3.548		
AY974351	Glycine max NAC3	0.048		−1.281	−1.197	3.152		0.865	−0.915
AY974352	Glycine max NAC4	−1.488				−1.241			
DQ028770	Glycine max NAC2	1.729	2.915	2.317		3.460	3.927	1.267	
DQ028773	Glycine max NAC5				3.248				4.993
DQ054363	Glycine max DREB2 gene		1.830	0.001			5.183	0.944	
DQ055133	Glycine max DREB3	0.464	0.989			2.405	2.232		
DQ055134	Glycine max C2H2		2.132		2.538		0.478		1.454
J01298	Glycine max ACT1	4.058				−0.377			
S15849836	DNA- binding protein	0.302			−0.424	−0.946			1.197
S15850208	Hunchback protein like		0.818		0.729		2.317		−0.205
S15940089	Zinc finger protein	0.523			0.755	1.789			−0.723
S21537216	MYB domain transcription factor			−0.024				0.953	
S21537821	SET-domain transcriptional regulator family	−1.771	−1.238		0.631	−0.613	0.449		−0.359
S21537971	Other transcription factor families		1.312	0.006	1.499		3.225	0.536	−0.177
S21538195	WRKY domain transcription factor			1.903	3.901			1.318	0.291
S21538405	Zinc finger protein	−0.342	−0.727			1.321	2.071		
S21538617	MADS box transcription factor	0.758				2.759			
S21539162	Other transcription factor families		0.569		0.630		2.007		1.227
S21539619	Other transcription factor families	−0.113	0.184		2.381	1.281	2.028		−0.025
S21539727	Homeodomain transcription factor		1.385	−0.126	0.849		2.802	−0.481	−0.659
S21540786	General Transcription	0.068			2.795	1.219			1.171

Table S1. *Cont.*

Accession No.	Genotype	PI408105-A				S99-2281			
	**Flooding Duration (Day)**	**1**	**3**	**7**	**10**	**1**	**3**	**7**	**10**
	**Gene Function**	**Log$_2$ (Flooded/Control) Expression Ratio**							
	Transcription factors								
S21565790	Putative transcription factor	0.030		−0.162	1.754	1.199		0.700	1.279
S21566054	G2-like transcription factor, GARP	−0.973	0.932			0.834	2.245		
S21566080	Zinc finger protein	3.057	2.773		−1.018	5.521	5.559		0.744
S21566748	Myb-related protein	−1.157		−0.709	−0.339	1.600		0.736	0.478
S21567785	WRKY domain transcription factor		−0.262				1.314		
S22951753	Hunchback protein like		−0.230				3.339		
S22951976	Aux/IAA				1.771				−0.921
S22952226	Trihelix, Triple-Helix transcription factor	0.421	1.202			1.912	3.142		
S22952239	NAC domain transcription factor		3.107				2.513		
S22953062	WRKY domain transcription factor	1.006				2.117			
S23061205	Leucine zipper transcription factor	−0.131	0.469	0.363	1.069	1.601	2.883	−1.308	−0.289
S23061430	LUG	−0.121			2.469	0.638			2.087
S23061455	Aux/IAA	0.158			2.435	1.452			−0.038
S23061550	bHLH,Basic Helix-Loop-Helix	−0.467	−0.442			0.862	1.171		
S23061947	Trihelix, Triple-Helix transcription factor			1.370	4.042			2.833	2.012
S23062231	Zinc finger protein	−0.938		−0.857		−1.091		−1.141	
S23064130	General Transcription			0.257				1.716	
S23064915	CCAAT box binding factor			0.350				0.668	
S23064932	MYB domain transcription factor				−4.083				−1.067
S23065007	Other transcription factor families	−0.579			2.454	1.235			0.222
S23066857	Bromodomain proteins			0.603				0.849	
S23067564	MYB domain transcription factor	−0.325			1.737	1.559			1.278
S23068300	Myb-related protein	−0.620			2.775	0.590			0.908
S23069233	Putative transcription factor	−1.569				0.071			

Table S1. *Cont.*

Accession No.	Genotype	PI408105-A				S99-2281			
	**Flooding Duration (Day)**	**1**	**3**	**7**	**10**	**1**	**3**	**7**	**10**
	**Gene Function**	**Log₂ (Flooded/Control) Expression Ratio**							
	Transcription factors								
S23070183	DNA binding protein	0.368	1.180	0.497		2.418	4.402	−0.837	
S23070418	C2H2 zinc finger		0.452				1.298		
S23070876	General Transcription		−0.319	0.268	1.800		1.580	0.118	0.382
S23071068	TCP transcription factor	−0.339	−0.443			1.976	1.097		
S23071477	bHLH,Basic Helix-Loop-Helix			−1.476				1.264	
S23071935	Other transcription factor families		0.518		0.460		2.186		0.976
S4863935	CCAAT box binding factor		−1.512		0.941		−0.104		−0.456
S4872880	Other transcription factor families		0.443				−1.089		
S4875111	Aux/IAA	−1.224	−0.689		−0.052	0.135	−2.075		1.134
S4875857	Zinc finger protein		−0.861	−1.518			−1.551	−1.236	
S4875903	WRKY domain transcription factor		0.867				−0.052		
S4876683	ARF, Auxin Response Factor			1.333				0.368	
S4877094	Zinc finger protein			0.114	0.846			−0.724	0.387
S4877491	MYB domain transcription factor	0.581	0.980	1.179	−0.190	1.343	1.303	−1.266	1.852
S4879817	Zinc finger protein				1.200				−1.148
S4884795	Putative transcription factor				−0.845				−0.006
S4885448	Other transcription factor families	−0.240				0.742			
S4885901	Putative transcription factor	0.040				1.140			
S4895927	Putative DNA-binding protein	0.808		0.116	0.750	2.167		−1.400	−1.112
S4897794	bHLH,Basic Helix-Loop-Helix				−0.290				−0.291
S4898613	Zinc finger protein		0.909	−1.728	−1.464		0.197	−1.669	−0.614
S4904584	WRKY domain transcription factor	0.574			1.207	−0.462			−1.730
S4908810	C2H2 zinc finger	−1.839			3.934	−1.411			0.486

Table S1. *Cont.*

Accession No.	Genotype	PI408105-A				S99-2281			
	**Flooding Duration (Day)**	**1**	**3**	**7**	**10**	**1**	**3**	**7**	**10**
	**Gene Function**	**Log$_2$ (Flooded/Control) Expression Ratio**							
	Transcription factors								
S4909265	Putative transcription factor			−3.057	−1.363			−0.230	0.708
S4910460	MYB domain transcription factor	−1.296	0.288	0.024	0.770	0.172	1.653	−0.712	−0.235
S4911235	Other transcription factor families				−1.345				−0.424
S4911726	Putative transcription factor	0.317	2.073		1.691	1.957	0.051		1.416
S4913507	Zinc finger protein		−1.483	−0.220	0.533		1.927	−1.486	−0.975
S4917467	Zinc finger protein				2.562				1.017
S4925034	Other transcription factor families	−0.164				1.477			
S4930680	DNA- binding protein			0.293				1.747	
S4932942	CHP-rich	−0.867				1.381			
S4948369	Zinc finger protein				0.507				−0.111
S4950242	DNA- binding protein		2.264		1.964		−1.619		2.162
S4967941	MADS box transcription factor		−0.291				1.078		
S4976159	AT-rich interaction domain containing transcription factor		−0.382	−0.011			2.255	−0.546	
S4980774	Chromatin remodeling complex subunit		0.888		1.419		2.390		−0.121
S5019221	Putative transcription factor				−2.426				−1.959
S5045942	Zinc finger protein	0.017			2.195	2.904			0.660
S5046001	MYB domain transcription factor		0.730				2.403		
S5075763	HB,Homeobox transcription factor			−0.236	−0.249			2.805	−0.107
S5076266	bZIP transcription factor			−1.199	−0.207			1.004	0.629
S5088770	Other transcription factor families		−2.117		−1.435		−1.263		−1.735
S5100831	Zinc finger protein		−0.035	−0.435			3.714	−1.108	
S5103646	Agamous like				−0.572				0.990
S5129107	Other transcription factor families	−1.323		−1.057	0.990	−0.262		−0.232	0.103

Table S1. *Cont.*

Accession No.	Genotype	PI408105-A				S99-2281			
	**Flooding Duration (Day)**	**1**	**3**	**7**	**10**	**1**	**3**	**7**	**10**
	**Gene Function**	**Log2 (Flooded/Control) Expression Ratio**							
	Transcription factors								
S5130128	DNA- binding protein	0.813	2.417			1.716	3.220		
S5142323	Other transcription factor families		1.490				2.035		
S5146158	bZIP transcription factor		0.604				1.100		
S5146255	Putative transcription factor				0.614				1.242
S5146307	Putative transcription factor		0.569		0.864		1.236		0.170
S5146871	Aux/IAA		0.191		0.738		−1.691		−0.236
S6675518	Putative transcription factor				3.069				−0.155
TC205627	bZIP transcription factor				−3.548				−0.929
TC205929	AP2 transcription factor like			−0.416				3.612	
TC206208	YABBY2-like transcription factor				0.713				−0.767
TC208789	MADS box transcription factor	0.980			2.547	2.862			0.617
TC209970	bZIP transcription factor		−1.412				0.850		
TC211088	Putative transcription factor				−0.494				0.267
TC211951	MYB domain transcription factor	−1.678				−0.127			
TC211971	AP2/EREBP, APETALA2/Ethylene-responsive element binding protein family	−1.321		−1.092		0.186		2.610	
TC214990	MYB domain transcription factor	0.564	1.163		0.662	2.393	2.688		−0.331
TC215913	MYB domain transcription factor	−1.224	0.435			1.100	1.097		
TC216048	Other transcription factor families	−1.738	−1.457		1.786	0.167	0.458		−1.080
TC216103	bZIP transcription factor		−0.256		1.036		2.423		−0.132
TC220047	Putative transcription factor	−0.910				0.027			
TC220597	WRKY domain transcription factor		0.995				1.397		
TC221650	bZIP transcription factor			0.804				1.283	
TC223128	WRKY domain transcription factor	−2.013				0.539			
TC225723	WRKY domain transcription factor		−0.38		−3.55		1.39		−2.5
TC232307	Putative transcription factor				−0.035				−1.381
TC232817	Putative transcription factor		−1.880		0.447		0.019		−0.128

Table S1. *Cont.*

Accession No.	Genotype	PI408105-A				S99-2281			
	**Flooding Duration (Day)**	**1**	**3**	**7**	**10**	**1**	**3**	**7**	**10**
	**Gene Function**	**Log$_2$ (Flooded/Control) Expression Ratio**							
	Anaerobic Genes								
AF079058	ADH1	8.426	8.502			10.171	8.911		
AY496909	Enolase	−0.597				1.642			
BE021140	LBD4		−1.127				0.839		
BE330134	ACC oxidase		3.159	0.814	3.233		1.071	0.360	2.050
BE661691	LBD40	6.013				7.553			
BI701220	Phosphoglucose isomerase	6.131				6.963			
BI969683	Aldolase			0.251				−0.881	
CK768393	LBD41	−1.335	3.718	0.963		4.095	−0.605	1.037	
D16455	XET partial	−0.337				1.013			
DQ273841	ACC synthase			1.497				1.351	
EU165371	ALAAT1	2.366			3.863	3.732			0.743
EU165372	ALAAT2	−1.241				1.384			
U47143	Hemoglobin GLB1	2.974	4.569	2.241	0.623	5.442	7.361	3.220	4.860

Table S2. To evaluate the effects of flooding on two soybean genotypes (PI408105A and S992281) over four different flooding duration (1, 3, 7 and 10 days), the data were statistically analyzed for multi-factorial analysis of variance using SAS. The flooding, genotype, and time of flooding were considered as fixed factors and the block was considered as a random factor.

**Source**	**DF**	**ANOVA SS**	**Mean Square**	***F* Value**	**Pr > F**
Genotype	1	2450.68	2450.68	0.11	0.7357
Flooding	1	296,196.68	296,196.68	13.77	0.0002
Duration	3	430,427.97	143,475.99	6.67	0.0002
Genotype x Flooding	1	17,645.34	17,645.34	0.82	0.3651
Genotype x Duration	3	155,933.79	51,977.93	2.42	0.0645
Flooding x Duration	3	274,530.40	91,510.13	4.25	0.0052
Genotype x Flooding x Duration	3	108,060.03	36,020.01	1.67	0.1702

Table S3. The primer sequences of the transcription factors, house-keeping genes and anaerobic genes used in the qRT-PCR study.

**Gene Name**	**Gene Function**	**Forward Primer**	**Reverse Primer**
	Soybean transcription factors		
AY974349	Glycine max NAC1	TGAGGCCTAATAGAGCAACCGT	CCAAAGCCTTCTTCACCCCTA
AY974351	Glycine max NAC3	ACCCCACAGATGAGGAGCTTTT	CCAAACACTGCCTTACCTGGAA
AY974352	Glycine max NAC4	CAGCGATTGATGAACGGTGTT	TTGGTCCAATCCAGTAACCCC
DQ028770	Glycine max NAC2	CGATCGAGAAACTGCAACCAA	CCTCCTCCGCTTTTCAGAATCT
DQ028773	Glycine max NAC5	ACCTCCCCAAAACTGCAAAGA	GTGAGAGGTGGCAAAGCAGAA
DQ028774	Glycine max NAC6	TTCTGGAAAGCCACCGGTT	AATCGGTCTTGCATCCCCTAG
DQ054363	Glycine max DREB2 gene	AGATGTCCGCCGATTCAATTC	AATGGAGTGTGTCGACGAGGA
DQ055133	Glycine max DREB3	AAGCCCATCCCTATGAAGCAC	GGAGTCTAATCTCAGCCACCCA
DQ055134	Glycine max C2H2	AGTTACCGAACCCAACCATGAA	TCTCATGTGCCCTCCCAAA
S15849836	DNA-binding protein	AATTCCCAAGAATCCCTTGC	CCCTCAGTTGGTGCTGATG
S15850208	Hunchback protein like	TGCACCCAGTTGTCATCAAT	TTGAGCAGCATCCAATCAAG
S15850391	Other transcription factor families	TGTGCTCTGAGGATCATTCG	GATGAAGAAGCCGAAGTTGC
S15940089	Zinc finger protein	AAACCACCCAAGGTGATCTG	TGTCGCGAATCGTATGAGAA
S18531023	Zinc finger protein	GAAGATGGCAAGGTCCTTCA	GATTGACCCCATTTGACCAC
S21537216	MYB domain transcription factor	GACCCATCACGAAAAGAGGA	AAAGCTGTTTGTGCAGAGCA
S21537821	SET-domain transcriptional regulator family	CAATGCTGCGTCTCACTTGT	CATACATGAATGGGGCCTCT
S21537971	Other transcription factor families	AGAGAGCCTTCCCATCTGCT	CATGTGCCTTGTACCGATTG
S21538195	WRKY domain transcription factor	GCATCGGCAAATACTTACACAA	CTTGGTCCCATTACTCAATCAA
S21538405	Zinc finger protein	CAGGAACAGACATGGCACTG	TGGACAGTTCCTCAGATCCC
S21538617	MADS box transcription factor	ATTTGACTTCTGGGGAGCCT	GACCCCACAAGAGCAAGAAG
S21539162	Other transcription factor families	TCGACCTCTCCAAATCTGCT	TTGTAAGTGGAAGGGGCATC
S21539619	Other transcription factor families	ATGGCCAATTGGAGTATTGC	GGACAACCAGTCAAGGGAAA
S21539727	Homeodomain transcription factor	ATTGCCATTTTCAAGCCATC	TGGAGCAACAGTACGCCATA
S21540786	General Transcription	ACAGCATCAACCTTAGCCGT	TTACACCCCAGCTGTTCCTC
S21540792	Zinc finger protein	CAAGGCTGACCCAAATCACT	TGTGGAGCACTGGTGTTTTT
S21565183	bHLH,Basic Helix-Loop-Helix	TTAAAAGCTGGCATTCTGCAT	CCAAACATGAATAGGACCCG
S21565790	Putative transcription factor	AAGTTGTATGGTTGGGCCTG	ATCCCCGCCTCATACTATCC

Table S3. *Cont.*

Gene Name	Gene Function	Forward Primer	Reverse Primer
		Soybean transcription factors	
S21566054	G2-like transcription factor, GARP	CCTTAAACGTTGCTTCCCAC	CTTGCAAATGCTGGGGTTT
S21566080	Zinc finger protein	TTACACTGTTGAACGCAGCC	ATGACCCTTTGAGCACAACC
S21566748	Myb-related protein	ATGCCCCTCTTCCAACTCTT	TCCTTTTGTTTTGGGGAATG
S21567785	WRKY domain transcription factor	CCATCACCTGATATCCCCAC	ATGACCCAGAGCCAAAAAGA
S22951753	Hunchback p+C73rotein like	CCTTGAGCTGAGTTCTGGCT	GGTTTTCATGATGACCCTGG
S22951976	Aux/IAA	GTCACCCAAGTAACCCACCA	AGGGCATTTTCTCATGCCTA
S22952226	Trihelix, Triple-Helix transcription factor	CTGCATTCTCTGCAACTCCA	TCTGAAATTCGGTGAGGCTT
S22952239	NAC domain transcription factor	ACACACAACACAGAACGACG	CTCGGGAATAATCAGATGTCG
S22953062	WRKY domain transcription factor	TTTACATTGCAACCACCACC	AAGAAAGGGGAACTGTTGGG
S23061205	Leucine zipper transcription factor	GAATTGCTCGGCTCATTTTC	TGAAGGCGAAGAGTCTGACC
S23061430	LUG	TCTTTGGGTGGAAATCAAGG	CGTTTGATACAACTGTGCGG
S23061455	Aux/IAA	CGCCATGACAACATAAAACG	GAAGCGAGAACTGAAGGCAT
S23061550	bHLH,Basic Helix-Loop-Helix	CTCCCGGATAGCTGATGAAA	TCAATGAATGCTCAACCTGC
S23061682	Alfin-like	CATCGTCATCTTGATCGTCC	AAGTCCAGCTCTAAGCAGCG
S23061947	Trihelix, Triple-Helix transcription factor	CGGTTACAATGGGCTTCTGT	CAGGCTGGTGATGTCATTTG
S23062231	Zinc finger protein	CCCACCAAGGTTTGTAATGC	GCAGCACCTGAAATTAGGGA
S23062909	bHLH,Basic Helix-Loop-Helix	ATCCAGGGCCATATTGTTGA	CTTCTTCGCTCGGAATGTGT
S23063261	Myb-related protein	GCCACTCCTTTCAGGAAGTT	CCCAAGTTCTTATGTGAATACCC
S23063489	C3H zinc finger	TTGAGGAACCACCACATTGA	CACCCTCTATGACGGAGGAA
S23064130	General Transcription	TCACAAGCCTTGCACTTTTG	TTGGAATGGGTGGTGAATTT
S23064915	CCAAT box binding factor	CCATGGCCCATAGTAAATCG	AGACACAATGCAAGAATGCG
S23064932	MYB domain transcription factor	AATCATAGCACCGCTCTTGG	GTAACCTGGAGGCTGTCCTG
S23065007	Other transcription factor families	CACGTCAGCAAACGTCAGAT	GGTTGTTTCCGACAAGGAGA
S23066857	Bromodomain proteins	CCAGCAGCACAACAGGAGTA	CCAGCACTGGTTGCATATTG
S23067564	MYB domain transcription factor	CCTCGTTCAGTGTCTTCTGCT	CATGATGAAACTGCTCAGGAA
S23068300	MYB-related protein	CAGCAGAACCGTTTGTTTGA	AGATAGAAGGCGATCCAGCA
S23069233	Putative transcription factor	AATCCCTTGAATTGGAACCC	TTCCAAGGACATCCAGAAGC

Table S3. *Cont.*

Gene Name	Gene Function	Forward Primer	Reverse Primer
		Soybean transcription factors	
S23070183	DNA binding protein	GAGCAAAGGATGTTTCTCCG	AAACAGCAGCCACAACACAG
S23070418	C2H2 zinc finger	TCCCTCGGCTCAAATATCAC	CCCTTAATAGGGTTGGGCTT
S23070876	General Transcription	GACCAATCATTCCAGGCATT	GCCGAGAGAGGACAAACAAA
S23070894	SBP,Squamosa promoter binding protein	GCACGTGTTGTTGGTTTTTG	TATGACTATGCATCCCTGCG
S23071068	TCP transcription factor	ACCAGCCTTTCCCAACTTTT	TCAGATGGGTTGGTGGTGTA
S23071477	bHLH,Basic Helix-Loop-Helix	CCTCGCATCGGAGTTATTGT	GAGTTTCAACCAGCAAAGCC
S23071935	Other transcription factor families	GCAACCTTGTTACCACTTCCA	TCTTGAGTTTCTTTCCGGTGA
S23072065	MYB domain transcription factor	TGGACCAGGAATATGCACAA	TCCCGAGACAGGATGAGAAC
S4861946	AP2/EREBP, APETALA2/Ethylene responsive protein family	AATGCAGTGTCTGCAACGAG	CCTCCCCATTTTCATGCTTA
S4862202	Other transcription factor families	CGAGTGAGAGCTCTGCTGTG	GAGCACTGGACTGCCTTACC
S4863935	CCAAT box binding factor	CCATGCAAGAATGTGTGTCC	AGCAAATATCGTCGCCATTC
S4864621	Other transcription factor families	GGGTAATTTGGGGGAAAAGA	TATGTTCCGTGGCGTACAAA
S4866988	Other transcription factor families	GCCATTTCTCTTAGGGGGTT	GGGAAAGGGGTTTCACAGA
S4867907	Putative transcription factor	CGCTTAAGTGGCCGTTTTTA	TTTTCGTTTTTACCAACCCG
S4869132	TUB transcription factor	CCAGCGCTGATTTGATGTTA	CCAGCAGAAAGCTCCAAAAC
S4870629	MYB domain transcription factor	TCCTTTCTCTTTTGGTGGGA	GGGTCCGTACAAGGAACAGA
S4872717	DNA- binding protein	GCTCTTCCGTGCTCACTTCT	GGCCGAAGATAATAGCACCC
S4872880	Other transcription factor families	GCAAATTCATGGAAGAGGGA	AATTGCTTCCTGGACCGTAA
S4875111	Aux/IAA	TGTTCACCTGCTGAAACTCG	CGCACCTAGCTTCATTCCAT
S4875857	zinc finger protein	GGGCTCGTAGGTAACGTCAG	GTCATAGCCGGCGAATTAAG
S4875903	WRKY domain transcription factor	TCAGGGATCCTCATCCTCAC	TGGATAATATTGTTGGCGCA
S4876683	ARF, Auxin Response Factor	TCTCTGTTGTTTCGCAGGG	GAAGTGAACTCCTTCGTGCC
S4877094	Zinc finger protein	GGGTTCCAAGAGATGGGAAT	GCGGCATAACACTTCTCTCC
S4877491	MYB domain transcription factor	TCCTTTTCTCTTCGCTTGGT	ATAACGGTGGCCTTCAGAAC
S4879817	Zinc finger protein	AGCTCAACACGACACACAGG	ACGATTTGACTTGGTTCCCA
S4882183	DNA- binding protein	GCTGAATCAAAGACGGAAGC	TCCGCTCACTCTTTATCCGT
S4882983	MYB domain transcription factor	GGTTCTGGGGTAACTACGCA	TCTACGTGGGTTGCAGACAG

Table S3. *Cont.*

Gene Name	Gene Function	Forward Primer	Reverse Primer
	Soybean transcription factors		
S4884782	RING zinc finger protein	CTAGTTCTCTCCTGGTGCCG	GGAAAACTTCATCACCCCAA
S4884795	Putative transcription factor	AGGCTGATCCATTTGGTTTG	CATCGATGATCCAGCACTTG
S4885448	Other transcription factor families	CTGCAAAGTTGTTGCTTGGA	TGGAGGATAACACATTCGCA
S4885901	Putative transcription factor	CTTCCTCAGGGAACAGTCCA	GAGAGGAGTCTTGGTGGTGC
S4888307	ARR	AGCCAGAGGCAGTGTGTTTT	CTGTTGCCAAGCACTCTGAA
S4891278	bHLH,Basic Helix-Loop-Helix	CTGAGTGATGCCATGGAGAC	CTGAACCCAACCATTCGTTT
S4891443	bZIP transcription factor	TTGCGTTTCAACCTCTTCCT	GGGATGGGAGGAGATTTGTT
S4891674	MADS box transcription factor	GATACTCCAGAACGGGACGA	GCTATGCTGATGCTCAGTCG
S4892093	AP2/EREBP, APETALA2/Ethylene-responsive protein family	GATCAACACCACCACCACAA	GAAGGGACTCACCGTTGCTA
S4895927	Putative DNA-binding protein	TGAGGGCCGTTTTGAGATAC	AGACCGACATTCCACCAGTC
S4896043	MYB domain transcription factor	GTTACTGGGAAGCAAGTGCC	TCAATTCCCAAGAAGAGAGCA
S4897794	bHLH,Basic Helix-Loop-Helix	TGATCGACGATATTCCCGTT	AACACCGACATTGGAAGGAG
S4898613	Zinc finger protein	CCATGGATGGAGCAGCTGTA	ATAACCAAGAAGCATTGCCA
S4900633	other transcription factor families	GAAACATGTATGAGCATCTGCC	CCCTCCCTCTACCTCACCTT
S4901375	EIN3+EIN3-like(EIL) transcription factor	CGGGCTTCTATCGTGTCATT	CTGATTACATGGGAGCACGA
S4901877	Other transcription factor families	TCACACACTCACATTCCGGT	GGTCCTTAAGTCATCAGCGG
S4904584	WRKY domain transcription factor	AATTCTGGCTCCGTGTTAGC	GCTCCCTTTAATGCCCTTCT
S4904949	RING zinc finger protein	GCGGGTTCTTTTCTGTTTTC	GAAACTCCCAAAAATACCTCAG
S4907367	MADS box transcription factor	ACCAATTCCCCCTTTGGGAG	AACGGCATTAATTTGGGGAC
S4908810	C2H2 zinc finger	GATCTCAACTTGCCAGCTCC	ACCCAATTGCTGCAGAGAAG
S4909265	Putative transcription factor	TGAGCCGAGAAAGAAAAGGA	TCACCTTAATCACTCTCACCGTT
S4910460	MYB domain transcription factor	TTCTCCAGTGTTCCCGTTTC	TGCAGTTGGTTTCAGCACTT
S4911235	Other transcription factor families	CCGAGTCGCGGTTAAAGTAG	TAACACAAGCAGATGCGACG
S4911726	Putative transcription factor	ATCACAATGCTTGGAGACCC	TGTGCTTGTCTGAGTCCTGG
S4913507	Zinc finger protein	GGAAGTAACAGCGTTGGAGG	CCCACTCATTCCCCTCACTA
S4914293	Zinc finger protein	GAAATACCAGATGGCAACCG	AAATCCTTGCATCAAGGTCG
S4917467	Zinc finger protein	CCCAACAAACATTACAGGAGTG	GAGTCAGCAATTGTCGTGTTG

Table S3. *Cont.*

Gene Name	Gene Function	Forward Primer	Reverse Primer
	Soybean transcription factors		
S4917546	MYB domain transcription factor	AATTCCTTGTGTGTCTCGGG	CTCACCCATGCTAGTGGGAT
S4925034	Other transcription factor families	TGACCGGGTTTCAGGAGTAA	TCTCCATCCATCCCTTTCTG
S4930680	DNA-binding protein	TGCATCAATTATCACGCACA	TGGTGCAATACGTAGCCTTT
S4932151	DNA-binding protein	CTGAGGAGGTGGCTCAGAAC	GCAGGTGATGTTGTGCAGTT
S4932942	CHP-rich	GGATCTCGCGAAACCGTTA	AGCCTAAGCCTCTCCACCTC
S4948369	Zinc finger protein	GAGGGAGATTTGTGAAGGCA	ACACACGAGCATTGAACTCG
S4950242	DNA-binding protein	GTTGCTGCTGCCTATGACTG	AACCGTTGTGTCCGGATTAG
S4967941	MADS box transcription factor	CATAGAGAACGCCACAAGCA	GAGCAACCTCAGCATCACAA
S4976159	AT-rich interaction domain containing transcription factor	CATGCAGAATAGTGGTCGCT	ACATGATTTCCGGGTCAACT
S4980774	Chromatin remodeling complex subunit	GGTGGCTCTTCTGATGCTCT	GGTCGAGATACAAAGCCTGC
S4981395	Other transcription factor families	CGGACGTCAAGAACACAAGA	ATTAGGCGTATTGGTGACCG
S4981647	ARF, Auxin Response Factor	ATGACATGACTCCACGATACG	CACCTATGCTGAATCTATCCACG
S4981738	Zinc finger protein	AGAGGAGCGAGTCCAATCTG	GAGTAACTGTGCGCAAACGA
S5002246	Other transcription factor families	TGCTGTTGGGTGAATGAAGA	GTTCTCAAAATCCATTGGCG
S5019221	Putative transcription factor	GAGTGGCAGGATAGTCCAGG	CTCTCTCCTTATCCGCTCCC
S5035170	EIN3 + EIN3-like (EIL) transcription factor	AAGACTGCCAGTTCACAGCC	CAAGAGATCTTCTTCTGCGAATG
S5045942	Zinc finger protein	CTAGCCACAAGAAGCCCAAG	CCATGCCACAAATTGAACAC
S5046001	MYB domain transcription factor	GAACATGCAAAGTGGGGACT	TTGTCATTCATGAGTTGCTGC
S5050636	NAC domain transcription factor	AGAATCGATACATGCGGGTT	GCAACTCACGGATCCTCGTA
S5075763	HB,Homeobox transcription factor	TGAACCATATCTAGAGACTACTACT	AGCATACTTCATACATAGGGCA
S5076266	bZIP transcription factor	CACCTGAGCCTAAGCCAAAG	GCATGGGCAAGAATTAGGAA
S5088770	Other transcription factor families	TTTAGGACAGTTGCTTGGGC	GAGAGTGTCGGGGATGTGTT
S5100831	Zinc finger protein	CGCGATGATTCTGGTTCTCT	GGAGAGGATTTCTACGGGCT
S5103646	Agamous like	ATGCTTTGGCCAATGTGAAT	TCTTCGTTGGCATGGTCATA
S5126262	MYB domain transcription factor	ATTTGTTGAGGCAGGAGCTG	AGGAAACCTGGTGCACAATC
S5129107	other transcription factor families	AAAACCTCTCTTGGCACGAA	TTTGAGTCTGCCTGGCTCTT
S5130128	DNA-binding protein	GTTCAGTGTTGCAGCCATGT	AACCTACCCAACGTAGCAAAA
S5146158	bZIP transcription factor	TCGTACCAGGTTCCACTTCC	GCAGCTTTCCAAAACCAGTC
S5146166	NAC domain transcription factor	TCTGGGATGATGATGTTGGA	CTTTGGTGTTGTTGCCAATG
S5146255	Putative transcription factor	TCTTCACCCCACAACAACAA	TAGGGAGTGGGAGTGGTGAC

Table S3. *Cont.*

Gene Name	Gene Function	Forward Primer	Reverse Primer
		Soybean transcription factors	
S5146307	Putative transcription factor	TGGGCTTCCTCAATTACACC	GTTGGGATACTGCATTGGCT
S5146871	Aux/IAA	GGCATTCTCGGAAATTGATG	CACCCCACCACTTGACTCTT
S6675518	Putative transcription factor	ACTCTAATGCTGCTGCTGGC	CTGCAGTGCCGAAATCTACA
TC205125	Homeodomain transcription factor	GCCGCCAGAAAGAAACTTAG	GCTTCGCCAAAGCTTGAATA
TC205627	bZIP transcription factor	CCGTCGTCTTCCTCTACTGG	GGGGGAAATGTTGGAGAAAT
TC205929	AP2 transcription factor like	TCCATGGGAAGTGGTAAGGA	GCCCGAATGTATCCAATGTT
TC206208	YABBY2-like transcription factor	TAATGATGAATCAGCTGCCG	TGGAGGCTTAGGGGTTTCTT
TC208789	MADS box transcription factor	GACCCCACAAGAGCAAGAAG	ATTTGACTTCTGGGGAGCCT
TC209970	bZIP transcription factor	AACCAACCCGTTTTTCAGTG	GAGAAGATTCACCCAGACGC
TC211088	Putative transcription factor	AGAGCTTGTGGAATTCCCTG	AGCATCCAATTCAAGGAACA
TC211951	MYB domain transcription factor	AGCAGTGGCAACAACAACAG	AGTTGAGGTGCTGGAAAGGA
TC211971	AP2/EREBP, APETALA2/Ethylene-responsive protein family	TCATCCATAAGGGTTGGAGC	GTCCATGTCTAAGGAGGGCA
TC214232	Cyclic-AMP-dependent transcription factor	CCAGAAGTTGGTCATGGGAG	TCAGAAACTCTTTGGCCCAT
TC214990	MYB domain transcription factor	TCCTGTCTTTTTGGTGGGAG	CGGGGTCTGTACAAGGAACA
TC215913	MYB domain transcription factor	TTTCATCAGGCAAAGCAATG	GCAGTGTCAGCTGCTTCATC
TC216048	Other transcription factor families	GATTCGCTCCATCATCACAA	GTGTTCCTCGTTGACGCTCT
TC216103	bZIP transcription factor	AGATGCGGTACATTTCGGAG	GGTTAGTGAGTCCAGCCGAA
TC216155	bZIP transcription factor	TCTTCTCCAGTGATCTCCGA	ATTGCACCAAGTGTGTCCTG
TC220047	Putative transcription factor	CCATGGATGCTGAGGAACTT	CTGCCACTTCATCCTTTGGT
TC220458	bZIP transcription factor	TCCAGCTTTGGAAGATCCAC	ATCCATCTCACTGCTTCCCA
TC220597	WRKY domain transcription factor	TAAGCTCCTGCCTTCCAGTG	GGTGCTTCTTGCAAAGGTTC
TC221650	bZIP transcription factor	CATGGAGCAACAAGCACAAC	GGAATCAGTGTGGCTCATCA
TC223128	WRKY domain transcription factor	ACGTACACCGGAGACCACTC	GAAGCAGGAGAGTGACCCAG
TC225042	Other transcription factor families	TCAACCCCTTCTCCTTCAAA	TTTTGGGTGGTGTTGGGTAT
TC225723	WRKY domain transcription factor	GCAAGCTTTTCTCTTTGGGA	ACTCACCCGCTTCAGTTCCT
TC232307	Putative transcription factor	GCGTGGGTATCACATGGTAA	CTCTCCCTCATCACTCTCCG
TC232363	MYB domain transcription factor	CTTTCGTTAATCGGTCATGAGT	CAATTGACCGACTAGTCACACC
TC232817	Putative transcription factor	CTCGTTTTTCTCGCTCGACT	GATCTTCCATGGACACGTCA

Table S3. *Cont.*

Gene Name	Gene Function	Forward Primer	Reverse Primer
		Soybean transcription factors	
U68763	Glycine max SCOF-1	ACATGGCTTTGGAAGCTCTCAA	ATGGTCGCGAGAACGCTTT
Z46956	Glycine max HSTF5	AACACAAAGCAAGCGCGTG	CACCCCAAAAAGCTTGGT CTT
		House-keeping genes	
AF475939	ELF1	CAGACTCGTGAACATGCTCTGC	TACCTGGCCTTGGAATACTTGG
D26092	Glycine max UBI-1	AGCTATTCGCAGTTCCCAAAT	CAGAGACGAACCTTGAGGAGA
D26092	Glycine max UBI-2	AGCTATTCGCAGTTCCCAAAT	CAGAGACGAACCTTGAGGAGA
J01298	Glycine max ACT1	CGTTAACTTTTCCCCTTCGCTC	CGAGGACGACCAACAATGCTA
M21296	Glycine max b-TUB1	ATGAGCGGAGTAACGTGCTGTC	CGAAGCCGACCATGAAGAAGT
		Anaerobic genes	
AF079058	ADH1	GGTTCGTCGACAATGAGCAATT	GCATGGTCACCTGGTTTCAGAT
AF079499	ADH2	TGGCCAAGTCATCAAATGCA	AACGCACTTCACCAGCTTGTG
AY496909	Enolase	ATCTCAATTGCATCGCTGCC	CATCGGAGCAAGTCAAATCCA
AY54729	Hemoglobin GLB3	AGTCTGCAACAGAAGGCCTCTG	CAAGGTTTGAAGGCCAAGCTT
BE021140	LBD4	AGTTGATGTGCTCCAAACCCA	GTGGTGAGAATTGGCGCATT
BE330134	ACC oxidase	AGAGCTCACACAGATGCTGGTG	ATGACAATGGAGTGGCGCA
BE661691	LBD40	CCAAGGTTGTGTGAAAATGCG	CTTGATCCATTGCAAACACGG
BI701220	Phosphoglucose isomerase	AGGTTGCATCCCTCAAGCAA	TGGAGCACGAAGAGCTACATGA
BI969683	Aldolase	TGACAAACATTCCCTTCTTGGC	CAAAAGTCCCTTTTGGTGCGT
BI970446	PDC3	AGCCA TCATTGTGCATCCTGA	TCACCCTCTTAGCCAACTCCCT
BQP224371	Glyceraldehyde3Phosphate dehydrogenase	CCAAGACCAATGGATCAACTCC	GCAGCGAAGGAAGTTTCTACCA
CK768393	LBD41	AAAACGTCTTGAAAGGCGCA	CTTTGGACACGTGGCGTATGT
D16455	XET partial	GAGTGAAATTCCCATTCGACCA	CGACCAATCCGTTTTCTCCA
DQ273841	ACC synthase	ATCGGAGATGGACATGGTGAAG	AAGACCCATTTGAATAACCCCG
EU165371	ALAT1	CAAACGCTTGCTTAATGCCAC	TCTGGAGGCAATATGGTGCAC
EU165372	ALAT2	TTGCAAGTGATCCACGACACA	AAAGGCTTTGGTGGCATGG
U00730	Cellulase U00730	GCTCATCCATCCCTTCAATCAA	CGCACCCACATGAGTATTTGG
U47143	Hemoglobin GLB1	CCATTTTAGAACCGGCGTAGC	TAGCCGGTGACCACATTTCTG
